# Thermometers

**Published:** 1886-01-20

**Authors:** 


### THERMOMETERS.

FAHRINHEIT'S SCALE, the one generally used in this country, makes the boiling point of water 212°, and places zero at 32° below freezing point.

The CENTIGRADE thermometer puts zero at the freezing point of water, and places the boiling point of water at 100°.

To reduce Centigrade degrees to those of Farinheit, multiply by 9 and divide by 5, and to this add 32. Then, to find the equivalent of 50° Centigrade in Fahrinheit :

$$50 \times 9 = 450$$
$$450 : 5 = 90$$
Add............32

